# The Asylums of Spain
†*Des Asiles d'Aliénés en Espagne, recherches historiques et medicales*. Par le Dr. Desmaisons, Directeur Medeein du Castel d'Andorte.


**Published:** 1860-04-01

**Authors:** 


### Art. III.—THE ASYLUMS OF SPAIN.†

To Spain belongs the honour of having first in Europe erected or set apart buildings for the special reception and protection of the insane. So early as the year 1410, a lunatic asylum was founded at Valencia, and one was established at Saragossa in 1425. Before the close of the century an asylum was also opened in each of the cities of Seville, Valladolid, and Toledo,—in the two former in 1436, in the latter in 1483. The foundation of these asylums was a work of the Church, and its execution was determined by feelings of compassion for the sufferings of the miserable insensates, who at that time were to be found in the streets of every city, a butt to the thoughtless and cruel provocations of the populace.

The events which more immediately influenced the formation of the first asylum, that of Valencia, the establishment of which gave the cue, so to speak, to the rest of the peninsula, are surmised rather than known. Dr. Morejon, in his bibliographical history of Spanish medicine, expresses the opinion, that the civil wars which desolated the kingdom of Valencia at the beginning of the 15th century, occasioned, from the suffering they gave rise

† *Des Asiles d'Aliénés en Espagne, recherches historiques et medicales.* Par le Dr. Desmaisons, Directeur Medecin du Castel d'Andorte.

to among the people, a large increase in the number of lunatics; and that the excessive and glaring misery of these unfortunates rendered necessary the foundation of the asylum. Dr. Desmaisons points out, however, that the civil dissensions to which Dr. Morejon refers originated at the death of King D. Martin I., which occurred in 1410, one year subsequent to the opening of the asylum.

The popular account of the origin of the asylum recites that one Juan Gilaberto Joffre, a brother of the order of Mercy, as he was wending his way to church in order to preach there, on the 24th of February, 1409, encountered in his way a band of children who were pestering an insensate. Grieved by the outrages to which the unhappy individual was exposed, the monk conceived the idea of exhorting the faithful to found a hospital in which lunatics could be received and protected, and this he did with such good effect as quickly to enlist the sympathies of the citizens, and to carry his object.

Dr. Desmaisons objects even to this account. He remarks that it is improbable that a novel institution, such as the first lunatic refuge at Valencia, could be, as it were, improvised—that it could result from a fortuitous circumstance. Seeking, therefore, for fuller information respecting Juan Gilaberto Joffre, he quickly found a confirmation of his doubts, and ascertained that the popular legend contained but a glimpse of the truth.

A monk of the order of Mercy, Fray Marcos Salmeron, in a work entitled, *Historical and Political Records of the Services which illustrious Members of his Corporation have rendered to the Kingdom of Spain*, and printed at Valencia in 1646, relates, in a notice of Juan Gilaberto Joffre, that he was eminent for his virtues and great eloquence, and that he had been particularly moved by the sufferings of the lunatics he saw wandering in the streets of Valencia. "He aspired without ceasing," writes the historian, "for the time when a refuge could be provided for them, in which they could be solaced and their sad malady cured, and his zeal caused him to seek ardently the means of realizing this project by charity." In depicting the brother Juan's great work as the consequence of thoughtful and constant meditation, the Spanish historian seems to give a much more likely account of the origin of the asylum of Valencia, than that which attributes it to a casual emotion, however lively that might have been.

Dr. Desmaisons adds to this statement a very ingenious suggestion. The Brothers of Mercy, of which association Juan Gilaberto Joffre was a member, were partly a religious, partly a military order, which had been founded at the Court of Arragon, and which was devoted to the redemption of Christian prisoners from the Infidels. To this duty the order added, by permission, the practice of physic—a privilege which the members exercised in common with the Brothers of Saint Jean de Dieu, long after the order of Mercy had been merged in other religious corporations. Now the avocations to which the Brothers of Mercy were devoted, bringing them into contact with the Mussulmen of Spain and Africa, perhaps more than any other charitable corporation, it is reasonable to suppose that they would become acquainted with the existence and organization of those hospitals specially destined for the insane, which had, even at that time, existed for ages in the East. It may be surmised, therefore, that from this source Juan Gilaberto Joffre and his brotherhood obtained their first notions concerning the sequestration of the insane, and that in the erection of lunatic asylums, the Spaniards, to use the words of M. Quinet, " copied yet cursed the spirit of Islamism at the same time." *

The success which had attended Juan Gilaberto Joffre's eloquence in Valencia was confirmed by the assent of King D. Martin I., obtained the 29th November, 1409, and in the year following letters apostolic, promulgated at Barcelona on the 26th of February, by Benedict XIII.,† (an Arragonese pope) authorized the construction of a chapel, and the appurtenances of the asylum, and the formation of a cemetery. For these purposes a house and garden were bought situated near the gate of Torrent, which has been since known as the Madmen's Gate—*la Porte des Fous.*

Seeing, therefore, a Spanish pope favouring an exclusively Spanish association for the care and treatment of lunatics, at the commencement of the 15th century, to Spain and the Spaniards must be ceded the honour of having organized the most ancient lunatic asylum of Europe.

Dr. Desmaisons, upon whose authority we give these details, does not himself, however, yield this credit to Spain, without a slight reservation in favour of France, which is supremely amusing from the *naïve* national vanity which it displays. Over the entrance of the Valencia asylum is, or was, engraved in the Limousin dialect, the inscription: *Spital de Nostra-Dona Sancta Maria del Innocents.* This recals to memory the intimate relations which, at the time when the inscription was written, existed between the south of France and Spain. Moreover, the true orthography of the name *Joffre* is doubtful. Historians are ignorant as well of the birthplace of the monk as of the condition of his family. But it is known that he visited the northern por-

* " Subjugués au dedans par l'esprit de l'Islamisme, dans le moment même où ils lui livraient au dehors une guerre acharnée, ils le maudissaient et le copiaient en même temps."—Edgard Quinet, Œuvres Completes, t. xi. p. 222. Paris. 1857.

† The antipape Benedict, the fiery Cardinal de Luna, relapsed from Charles VI. of France, of whom the remembrance cannot be separated from the history of insanity.—Desmaisons.

tion of France, in the exercise of his sacred duties, and that there his eloquence was attended with success. From these circumstances Dr. Desmaisons thinks that "he is authorized to suppose, at least he loves to believe, that in this matter France has given to Spain, in the Brother Jean Gilbert Joffre, a worthy successor of Français Pierre Nolasque, the founder of the order of Mercy!" Is France so poor in men who have discovered a large outlet for their charity in ameliorating the state of lunatics, that even one of her physicians can begrudge to Spain a solitary honour in this respect, on grounds so slight as to be all but visionary?

Not only, however, does Spain claim the credit of having created the first lunatic European asylum, but it would appear also as if to her was due also the first introduction of these institutions into other countries of Europe. The earliest special asylum for the insane in Rome, and probably in Italy, was, we learn from Cardinal Morichini, founded in 1548, and that it was the work of three Spaniards, Fernando Ruiz, Chaplain of Saint Catherine *dé Farrari*, and Diego and Angelo Bonne. Towards the close of the 14th century, the leper-houses of Italy had, as they became less encumbered by the diminution of leprosy, been made use of for the sequestration of lunatics. But when, at the termination of the 15th century, syphilis broke out for the first time or appeared in a greatly aggravated form, this custom was put an end to. The claim then which Italy advances to the honour of being the first European country in which special lunatic asylums were instituted must be set aside in favour of Spain. "Italy," writes Dr. Desmaisons, "offered assistance to the lunatic in hospitals destined to the most disgusting affections, or accorded aid with extreme parsimony, when Spain had opened asylums for these unfortunates." (p. 112.) When Italy had founded establishments for the insane, doubtless her example chiefly contributed to their being adopted in other countries of Europe, but Italian authors have erred in asserting that the lunatic asylums of Italy were the first refuges of the kind in Europe. These authors have unwittingly taken the first date of some of the leper-houses or other charitable institutions subsequently transformed into lunatic asylums, as the original date of the secondary use to which the buildings had been put, and thus fallen into error. It may easily be shown, Dr. Desmaisons tells us, that, for example, the Hospital of Bonifazio at Florence, the origin of which dates from the 14th century, was not made use of, in any of its dependencies, to receive the lunatics of Tuscany, until four centuries after its foundation.

If Spain began the good work thus well, she has lost her vantage-ground over Europe, and until very recently she has lagged greatly, and she still lags in the rear. Although, according to F. Marcos Salmeron, Juan Gilaberto Joffre had in view the cure as well as the protection of the insane, when the asylum of Valencia was established, yet, as we have already said, the association which he formed to carry out his views was actuated chiefly by charitable feelings. Hence we find that in 1414 permission was sought from Pope Ferdinand XIII., and in 1414 and 1416 from the kings of Arragon, D. Ferdinand I. and D. Alonso V., that the association might act in concord with the members of other societies, in performing additional charitable offices, such as burying bodies which had been abandoned, and accompanying criminals to the scaffold. These duties, however meritorious they might be, and particularly in the midst of the bloody civil strife that then prevailed, had the effect of distracting the disciples of Jóffre from the special mission which at first they had adopted. Thus at the very beginning the work of charity in favour of lunatics was nipped in its growth. The special character of the asylum being lost, its subsequent development was shackled, and in 1512, the primitive buildings of the Valencia Asylum having been destroyed by fire, the ancient house of the Innocents was annexed to the general hospital of the city. From this moment the progress of the asylum was arrested. It suffered from the heart-burnings which existed between the lay and clerical elements of the government of the hospital—this being divided between the chapter and municipality of the city. In 1785, Charles III. gave a preponderance to the laity in the superintending committee of the hospital, but the lunatics gathered no advantage from this change, from that habit of economizing at the expense of the patients, by diminishing as much as possible the number of officers and attendants, to which all municipalities, as Dr. Desmaisons well remarks, seem to be "instinctively carried."

Since 1849, the direction of the lunatic department of the hospital has rested of right, and in principle, with the central government at Madrid, and of the other departments with local committees. It does not appear that the insane have derived much benefit from this change, but rather that the defects in the organization of the asylum have become more apparent than when it was entirely under a provincial administration.

For the rest, the present building is defective in its structure, as is the case with all the older asylums, and contracted courts and edifices, and want of surrounding land—faults almost inseparable from a hospital constructed within a crowded city—interpose a bar to the effective treatment or the proper care of lunatics.

It is needless to describe in detail the causes which led to the decadence or comparative barrenness of results in other ancient lunatic foundations of Spain. The asylums were charitable refuges or prisons, and defects of construction and government led to similar evil consequences and abuses as occurred in the older lunatic establishments of the rest of Europe. A few facts of interest respecting the Spanish asylums may, however, be noted.

The founder of the asylum of Valladolid, D. Sanche Velasquez de Cuellar, in his testament dated the 13th February, 1436, formally excludes from participating in the benefits of the building those suffering from the dementia of old age. It is curious to observe in the very infancy of European asylums, a founder thus obviating in no small degree one of the chief causes which neutralizes the utility of our great public asylums at the present day, the encumbrance of the wards with incurable cases.

In 1849, the ancient asylum of Valladolid was vacated, the patients being transferred to a handsome mansion, surrounded by extensive gardens, outside the city. In 1840 the primitive building, which was situated in an insalubrious locality within the city, and utterly insusceptible of improvement, contained twenty-four patients. At the close of 1852, the number of lunatics in the new asylum had increased to 213—147 males and 66 females! This is a marked illustration of the advantages which a community derives from the better administration of its lunatic asylums.

It is worthy of remark, that the provinces pay five reals per day for each patient sent by them to the Valladolid asylum. But if the number of lunatics from any province exceed six, the amount of payment is diminished to four or even three reals per day, the latter being the minimum rate. This arrangement is thought to encourage the sequestration of pauper lunatics when needful.

The ancient asylum of Saragossa was destroyed by fire in the night of the 4th August, 1808, during the siege of the city. The archives, valued at upwards of 200,000*l.*, were lost in the flames. The present asylum was erected in 1819, and it forms a dependency of the general hospital. The building approximates in character to a gaol rather than a hospital, and is exceedingly unfitted to effect the objects for which it was constructed.

A train of the lunatics detained in the asylum of Saragossa still assist, or at least did so very lately, in the great religious solemnities observed in the city. The unhappy patients are clad in a habit partly green and partly brown, and a bib is suspended from the neck in front. They are accompanied by a drummer, who beats his instrument as they advance, and they are preceded by a banner, of which the colours, blue bordered with brown, signify symbolically *patience in adversity*.

The Saragossa asylum is devoted to the care of lunatics of the wealthier classes, as well as of those who are indigent. This, indeed, would seem to be the usual arrangement of the Spanish asylums. It is also the approved one, judging from the rescript for the model asylum at Madrid, to which we shall presently have more particularly to refer.

Dr. Desmaisons condemns strongly the custom of receiving the two classes of patients into the same asylum, and under the same roof. He considers that under such circumstances it is almost impossible to make the arrangements which are requisite for the proper occupation of the lunatics, with due regard to their social condition and previous habits of life. We cannot either in the in- or out-door exercises assimilate the duties assigned to the higher and lower classes of patients, while by placing them under one roof we are apt to contract the space which should be at our disposal for each class, without any advantages being obtained which at all compensate for the inconveniences and discomforts to which the system gives rise, and for the injury which the defects arising from it may occasion to the patients. From the evidence given as to the working of the chartered asylums in Scotland, before the Parliamentary Committee on Lunatics which sat last session, it would seem as if an almost similar opinion might be expressed with regard to those institutions, notwithstanding that they have all the advantages which skill and money can afford to them,* while this is far from being the case in the Spanish asylums.

It is interesting to remark that the number of lunatics in the Saragossa asylum were the same in 1859 as 1853. We have already noted the great increase in the number of patients in the Valladolid asylum coincidently with the introduction of great improvements as well in the housing as in the care of the insane. A similar occurrence has also been observed in connexion with a considerable amelioration of the condition of the insane in the asylum of Sancta Cruz at Barcelona. From this Dr. Desmaisons argues that the opinion of the rarity of insanity in Spain,

* "1360. Does it (the state of the chartered asylums) give satisfaction both to the friends of the poorer and richer patients to have them confined in one building, although not associated together?—I think that there are many disadvantages in having them under the same roof, as they are at Dundee.

"1361. What disadvantages do you refer to?—I think one of the principal disadvantages is, the requiring to have separate airing courts for both classes. They are grouped round the asylum, and they are much smaller than they would otherwise be, from the necessity of having two sets of airing courts.

"1362. They do not meet even in their amusements?—In any holiday amusements they meet, but not in the everyday routine of the asylum.

"1363. What other disadvantages have you to mention?—Private patients do not generally like being under the same roof with paupers.

"1364. Is there any unwillingness on the part of the relatives of the richer patients to place their relations in the same asylum with pauper patients?—I do not think there is when the two buildings are separate."—*Examination of Dr. J. Coxe, one of the Scotch Lunacy Commissioners.—Report*, 1859.

which has been expressed by several writers of authority, is erroneous. In so far as this opinion was founded upon the paucity and slight variation in number of the cases in the Spanish asylums, the recent experience of the asylums of Valladolid and Barcelona clearly show that this is to be regarded simply as a measure of the unfitness of those asylums for the reception and treatment of the insane. As the state of the Spanish asylums approximates more and more in character to those of central Europe, France, and England, in all probability the seeming comparative freedom of Spain from insanity will vanish.

The present asylum of Toledo was erected in 1793. It is a noble building in all its details except those which more immediately appertain to the uses for which it was designed. In this respect the interior arrangements are highly defective and unsuited for the treatment of lunatics.

In Madrid the insane are cared for in the general hospital and in a small asylum, furnishing accommodation for about seventy patients, at Legana, in the environs of the city.

According to a Spanish authority, D. P. Rubio, the number of insane under care in the different asylums of Spain, averaged, in 1847, *one* only in every 7462 inhabitants. The same author estimates that the proportion of lunatics existing in the kingdom is *one* in every 1667 population.

Spain has recently become conscious of the fact that of late years in her care of the insane she has lagged far behind the other Christian nations of the continent. She now seeks to regain a position more befitting the people who first in Europe made the lunatic an object of special charitable treatment. On the 28th of July last, a report on the present condition of the Spanish asylums was addressed to her Majesty of Spain, by the Minister of the Interior, Don Jose de Possada Herrara. From this report we learn that all the existing asylums need great and costly reforms and considerable sacrifices on the part of the State, and none more so than the asylum at Legana. The pettiness of the general arrangements of the building, its absolute want of water, and its defective situation and construction, render it unworthy of being the general establishment for the central provinces of the Monarchy. We are told, likewise, that the sad spectacle which has been presented for so long a time in the mad-houses of the kingdom, in which all classification of the patients is impossible, and in which no other treatment than perpetual seclusion, chastisement, and isolation can be generally had recourse to, is inconsistent with the honour of the nation, and can no longer be permitted, medicine having shown the practicability of a better and more commendable state of things and one more consistent with humanity. We learn further, that the law governing the beneficent establishments of the kingdom, sets forth that six public asylums shall be founded for the better care and treatment of the insane in different parts of the kingdom. It is intimated also, that the subsequent use, as a matter of economy, of the ancient asylums, as places for the care of lunatics, will be determined by the nature of the hygienic conditions and architectural arrangements of the buildings. Moreover, it is suggested that in fulfilment of the law already referred to, an asylum should immediately be erected at Madrid, which might serve as a model for the other provinces of the kingdom, when called upon in their turn to found asylums. The Minister finally submitted to Her Majesty a scheme for the construction of a model asylum, and it was decreed that this plan should at once be carried into effect.

The scheme thus submitted to Her Majesty and now promulgated is to be regarded as the culmination of Spanish notions on the construction of lunatic asylums, and as such it possesses considerable interest. Before, however, proceeding to describe the scheme, we would remark that if to the Church is to be attributed the first establishment of lunatic asylums in Spain, so also to the Church is probably to be assigned the cause of the unsatisfactory state of the asylums of Spain at the present day. Medicine, by no means, if we are to repose faith in the statements of travellers, holds an eminent social position in that country. Not only is the science at a discount with the people, but it is hampered and down-trodden by the priesthood. The former may be fairly assumed to have been chiefly influenced by the latter, and the latter have brought the full energy of ecclesiastical power to bear in cramping the energies of the doctor. We read of comparatively recent laws preventing the right teaching of medicine. In the bickerings between physic and theology, the former always must go to the wall, and even if the power of the priesthood has been checked in the administration of lunatic asylums by the strong hand of the law, physic has fared but little better for it, for then it has been brought into contact in this matter with the prejudices of the people, as represented by municipalities. But we need not go to Spain for examples to show how often the prepossessions of corporations or local governments of any kind act most fatally upon the utility of lunatic asylums. It would not perhaps be difficult to prove that to the unfortunate tone assumed by the Church in Spain towards physic is chiefly to be attributed the fact that the Spaniards have taken a longer time than any other Christian European nation to learn that a lunatic asylum should be a hospital for the cure, not a prison for the detention of the insane—that it should be a medical institution, not merely a charitable refuge. There are many able and accomplished Spanish physicians who have long seen with grief the state of their country's asylums, but whose efforts for their amelioration have been futile from their inability to incite local authorities to action,—for the success which has attended the efforts of Dr. D. E. Pi y Molist at Barcelona stands almost alone, the improvements at Valladolid being the work of the director of the asylum, a priest, whose excellent example has been but too rarely followed. The medical profession of Spain is guiltless, we believe, of the disgraceful state which still, according to the official account, characterizes the majority of the Spanish asylums.

To return to the scheme proposed by the Minister of the Interior. The Royal Decree instituted a public competition for the erection of a model asylum, or *Manicomia,* as it is termed, the plans to be designed in accordance with the subsequent programme and to be presented within ninety days after the promulgation of the decree.

The programme states that the population of the asylum would consist of 500 lunatics of both sexes, and a due number of officials and attendants, and sets forth the following requisites:—

The establishment to be divided into two principal and independent sections: the one to contain 250 women and the other an equal number of men.

Each section to be sub-divided into two divisions: the first for lunatic-boarders of the first and second class; the second for paupers.

The *boarders' division* to have two quarters: the first for tranquil lunatics; the second, for restless and filthy.

The *pauper division* to have four quarters: the first for tranquil lunatics; the second for the restless and dirty; the third for children and old people; the fourth for criminals. This division also to have attached to it an infirmary for the treatment of accidental or occasional maladies.

The 250 lunatics to be estimated thus:—

| | | | |
|---|---|---|---|
| Boarders' division | First class | 40 | 100 |
| | Second class | 60 | |
| Pauper division | Adults | 100 | 150 |
| | Children and old people | 40 | |
| | Criminals | 10 | |

The distribution of the lunatics to be arranged as follows:—

| | | | |
|---|---|---|---|
| *Boarders.*—Quarter for the calm | 80 | First class | 30 |
| | | Second class | 50 |
| Quarter for the restless and dirty | 20 | Restless, first class | 5 |
| | | Dirty, *id.* | 3 |
| | | Restless, second class | 8 |
| | | Dirty, *id.* | 4 |

| | | |
|---|---|---|
| *Paupers.*—Quarter for the calm | . . . . . . . . . | 86 |
| Quarter for the restless and dirty | . . . | 30 |
| Quarter for children and old men | . . . | 24 |
| Quarter for criminals | . . . . . . . . | 10 |

Dispositions to be made for 20 restless and 10 dirty lunatics.

The general appurtenances of the asylum were to be regulated in the following manner :—

I. The entrance to the asylum to have, (1) a spacious vestibule ; (2) an apartment for the porter ; (3) a waiting-room.

II. On the ground-floor, near the entrance, to be placed (1) the quarters of the porter; (2) the apartments of the resident physician, consisting of reception-room, cabinet, bed-chamber, and two or more other rooms; (3) another compartment for the manager, with rooms for officers.

III. A hall to be designed and decorated with care for important receptions and for the commission ; also—

IV. A chapel, so situated as to render it easily accessible to the lunatics from all quarters of the asylum, and arranged so that they may take part in the religious exercises in places conveniently separated.

V. As near as possible to the apartments of the resident physician, must be, (1) a room destined for a library ; (2) another to serve for a museum of pathological anatomy and phrenology, and as a cabinet for physical and surgical instruments ; (3) an amphitheatre, light and airy, capable of containing 150 persons; (4) a room for dissection, anatomical studies, autopsies, and experiments.

Required also—

VI. (1) A dispensary ; (2) a chemical laboratory ; (3) a closet for the principal dispenser ; (4) rooms for his assistants during their daily attendance ; (5) places for pharmaceutical stores.

VII. (1) Pantry; (2) cellars for the preservation of food and liquids ; (3) one or more poultry-yards ; (4) a skinning-yard ; (5) a corn-mill moved by horses, with its dependencies.

VIII. A depository, a general magazine for linen and clothing, composed of two rooms, in addition to one for the clerk ; (2) a second, for mattresses and other objects of similar usage ; (3) a wash-house, and all its appurtenances; (4) another wash-house, for the use of the boarders and the *employés* of the establishment ; (5) rooms for the finishing and mending of the linen and clothing.

IX. Two halls for medical gymnastics.

X. Coal and fire-wood depots, so placed as to avoid all danger of fire.

XI. Coach-houses, squares, shady places, parterres, kitchen-gardens, covered walks.

XII. *Apartments:* (1) For the presiding physician; (2) two assistant physicians; (3) two almoners; (4) the dispenser; (5) the manager; (6) six clerks; (*employés d'Administration*); (7) two chief hospital attendants; (8) four second-rank ditto; (9) a doorkeeper; (10) ten porters; (11) twenty attendants of both sexes; (12) twenty servants, as gardeners, watchmen, for the use of wash-houses, &c.

XIII. A cemetery.

XIV. The portion of the edifice destined for the lunatics to have but a ground and first floor; this latter to be surmounted with a second, if needful, for the apartments of attendants and servants.

XV. Sinks, conduits, wells, pools, troughs, and reservoirs of water to be conveniently distributed.

The *appurtenances of the sections* to be as follows :—

Each section to possess, (1) a vestibule; (2) a reception hall; (3) a room for the porter of the section; (4) a consultation cabinet for the physicians; (5) an apartment for the chief attendant; (6) kitchen, with its necessary dependencies; (7) refectory for the attendants and other servants; (8) gardens, covered and open walks, and courts corresponding to the section.

The *appurtenances of the divisions* to be in this wise :—

In each division (1) a reception hall; (2) a room for the porter; (3) a *lingerie;* (4) a depository for linen and soiled vestments; (5) another for the utensils and other things belonging to the division; (6) an apartment for the clerk charged with the care of the clothing and furniture.

*Boarders division.—Male section:* (*a*) *Quarter for calm lunatics.* —In this quarter to be arranged: (1) a reception hall; (2) a parlour; (3) thirty residences for boarders of the first class, and fifty for those of the second. The residences or pavilions of the first-class boarders to be composed of a vestibule, a drawing-room, a closet with alcoves, a dining-room, a dressing-room, and a small bed-chamber for the attendant or domestic. Those for the second-class boarders to consist of a parlour, a bed-chamber with alcoves, a dressing-room, and a bed-chamber for attendant or domestic. Also, (4) a refectory for those who might wish to take their meals together; (5) a common room; (6) a room for billiards and other games; (7) a reading-room; (8) six bath-rooms.

(*b*) *Quarter for restless and dirty lunatics.*—This quarter to be subdivided in such a manner that the residences destined for the dirty patients shall be separate from those set apart for the restless.

Required here: (1) A reception-room; (2) a parlour; (3) twenty residences arranged in the same mode as those for the calm patients. Of this number, six to be set apart for boarders of the first class, and fourteen for those of the second; (4) the apartments destined for the dirty patients to be arranged alike for the first and second class boarders; (5) four bath-rooms; (6) a common room, adjoining which must be a room for the attendants.

*Boarders division—Female section: (a) Quarter for calm lunatics.*—This quarter to have: (1) a waiting-room; (2) a parlour; (3) the same number of apartments as in the male section, and arranged in the same manner; (4) a refectory for the patients who wish to take their meals together; (5) a recreation hall; (6) a work-room; (7) six bath-rooms.

(*b*) *Quarter for restless lunatics.*—The same arrangement to be made in every respect as in the quarter for restless and dirty males.

*Pauper division.*—(*a*) *Quarter for calm lunatics.*—Each section of this division, *male* or *female*, to have the following arrangement:

(1) A reception hall; (2) a parlour; (3) dormitories to contain twelve, eight, six, and four beds, and chambers for single beds. The beds to be placed at least six feet the one from the other. (4) Apartments contiguous to the lunatic dormitories to serve as day and night rooms for the attendants, and so planned that they can exercise a complete surveillance over the patients; (5) water-closets; (6) a refectory; (7) a school-room; (8) work-shops; (9) a common room; (10) an infirmary consisting of two rooms, one to contain twenty beds for medical cases, the other, ten for surgical; (11) a neighbouring closet for the physician; (12) another closet, well lit, for surgical operations; (13) two rooms for the assistant-physician and superintendent nurse; (14) eight bath rooms.

(*b*) *Quarter for the restless and dirty.*—Each of these quarters (male or female) to have:—

(1) A reception-room; (2) a parlour; (3) twenty cells for the restless and furious, consisting each of a room and of an alcove so disposed as to facilitate surveillance; (4) ten cells for the dirty lunatics, formed also of a room and an alcove. These cells to be removed as much as possible from the twenty first named. (5) Apartments for the attendants from which they can observe the restless patients without being themselves seen; (6) water-closets; (7) a common room; (8) a work-room; (9) the same number of bath-rooms as for the calm patients.

(*c*) *Quarter for children and old men.*—This quarter to be arranged in a similar manner to, and to have dependencies like those of, the quarter for calm lunatics, regard being had to the number of individuals already estimated for this category.

(*d*) *Quarter for criminals.*—This quarter to consist of:—(1) an apartment for the porter; (2) a parlour; (3) ten prison-cells (*cellules de sûreté*) having no communication with one another, and two sets of two or three rooms; (4) apartments for the keepers disposed so as to secure a rigorous surveillance; (5) a common-room; (6) another room arranged for the physicians'

examinations and for the reception of declarations; (7) a garden or court-yard for the promenades of the prisoners.

This is the scheme issued by the Minister of the Interior, and it is the latest phase of action in Spain on lunacy questions. The model Manicomia is to be constructed on an estate the superficies of which amounts to about 100 *fanegas*. The *fanega* is a measure of 400 square fathoms arable, and of 500 pasture land.

---

### Art. IV.—THE MENTAL PHILOSOPHY OF FICHTE THE YOUNGER.*

